# TRIM67 Suppresses TNFalpha-Triggered NF-kB Activation by Competitively Binding Beta-TrCP to IkBa

**DOI:** 10.3389/fimmu.2022.793147

**Published:** 2022-02-22

**Authors:** Wenchun Fan, Xueyan Liu, Jinyan Zhang, Liuxing Qin, Jian Du, Xiangmin Li, Suhong Qian, Huanchun Chen, Ping Qian

**Affiliations:** ^1^State Key Laboratory of Agricultural Microbiology, Huazhong Agricultural University, Wuhan, China; ^2^Division of Animal Infectious Diseases, College of Veterinary Medicine, Huazhong Agricultural University, Wuhan, China; ^3^The Cooperative Innovation Center for Sustainable Pig Production, Huazhong Agricultural University, Wuhan, China

**Keywords:** TRIM67, TNFa, NF-kB signal pathway, beta-TrCP, IkBalpha

## Abstract

The transcription factor NF-κB plays an important role in modulation of inflammatory pathways, which are associated with inflammatory diseases, neurodegeneration, apoptosis, immune responses, and cancer. Increasing evidence indicates that TRIM proteins are crucial role in the regulation of NF-κB signaling pathways. In this study, we identified TRIM67 as a negative regulator of TNFα-triggered NF-κB activation. Ectopic expression of TRIM67 significantly represses TNFα-induced NF-κB activation and the expression of pro-inflammatory cytokines TNFα and IL-6. In contrast, Trim67 depletion promotes TNFα-induced expression of TNFα, IL-6, and Mcp-1 in primary mouse embryonic fibroblasts. Mechanistically, we found that TRIM67 competitively binding β-transducin repeat-containing protein (β-TrCP) to IκBα results inhibition of β-TrCP-mediated degradation of IκBα, which finally caused inhibition of TNFα-triggered NF-κB activation. In summary, our findings revealed that TRIM67 function as a novel negative regulator of NF-κB signaling pathway, implying TRIM67 might exert an important role in regulation of inflammation disease and pathogen infection caused inflammation.

## Introduction

The nuclear factor kappa B (NF-κB), an important early transcription regulator, is involved in various cellular responses to stimuli, such as ultraviolet irradiation, heavy metals, cytokines, free radicals, and microbial infection (1). NF-κB plays a crucial role in many cellular events, including inflammation, cancer, cell growth, apoptosis, and immunity (2–4). In resting state, the NF-κB complex is maintained in the cytoplasm in an inactive form through inhibitor IκB proteins. Upon stimulation, IκB proteins are phosphorylated by IκB kinases (IKK) complex such as IKKα, IKKβ. The phosphorylated IκB proteins are degraded by 26S-proteasome pathways (5–7). With the degradation of IκB proteins, NF-κB is freed to be transported into the nucleus, where it activates the transcription of a large number of genes (3).

Ubiquitination regulates the activation of NF-κB signaling pathways in different stages (5). β-transducin repeat-containing protein (β-TrCP) is a subunit of the host SKPI-CUL1-F-box proteins (SCF) E3 ubiquitin protein ligase complex, which subjects their substrates to degradation through proteasome pathways (8). Hakakeyama discovered that β-TrCP is associated specifically with phosphorylated IκBα. β-TrCP recognizes the phosphorylated IκBα and rapidly mediates its ubiquitination and degradation, to induce the nuclear translocation of NF-κB (9). Liang also found that β-TrCP mediates the phosphorylated p100 undergoing ubiquitination and degradation to generate p52, resulting in the nuclear translocation of NF-κB2 (10). Hence, β-TrCP plays a crucial role in the activation of NF-κB signaling pathways.

Tripartite motif-containing (TRIM) proteins constitute a superfamily and share a conserved motif architecture known as RBCC: RING finger domain, one or two B-box domains, and a coiled-coil domain. The C-terminus of TRIM proteins is variable and is similar to the NHL, ARF, PRY/SPRY (B30.2), and other uncharacterized domains (11). TRIM proteins are involved in many cellular processes, including inflammation, cancer, autophagy, and immunity (12–17). Emerging evidence suggests that TRIM proteins are important in the regulation of NF-κB activation. TRIM30 alpha and TRIM38 are well characterized as inhibitory regulators for NF-κB activation as they target TGF-β–activated kinase 1 (TAK1)-binding protein 2/3 (TAB2/3) for degradation (18, 19). TRIM13, an endoplasmic reticulum(ER) membrane anchored E3 ligase, interacts with NF-κB essential modulator (NEMO) and regulates ubiquitination, thereby inhibiting TNFα-triggered NF-κB activation (20). TRIM9 and TRIM39 were identified as novel negative regulator for NF-κB activation. TRIM9 hijacks β-TrCP to block its mediated degradation of IκBα and p100, thereby inhibiting canonical and non-canonical NF-κB pathways (21). TRIM59 targets ECSIT to negatively regulate NF-κB signal pathway (22). However, the potential capability and mechanisms of NF-κB regulation of other TRIM proteins, such as TRIM45, have not been fully understood (23).

TRIM67, a member of the TRIM protein family. Currently, minimal information is known about TRIM67, although it is recognized to be capable of negatively regulating Ras activities by targeting 80K-H for degradation and then triggering neuritogenesis (24). In recent studies, TRIM67 has been reported that it’s playing an important role in cancer development (25–28) and brain development (29–32). In this study, we performed a microscopic observation to investigate the effects of 22 TRIM proteins on the TNFα-induced nuclear translocation of p65. We found that TRIM67 negatively regulates TNFα-triggered p65 nuclear translocation. Further studies, we identified β-TrCP as a TRIM67 interaction protein through immunoprecipitation combined mass spectrometry. Finally, we found that TRIM67 exerted no effects on the β-TrCP protein level change but competed with IκBα for β-TrCP binding to inhibit β-TrCP -mediated IκBα degradation. Thus, in this study, we demonstrate that TRIM67 as novel regulator of NF-κB signaling pathway that suppressing TNFα-triggered NF-κB activation by interrupting β-TrCP-mediated IκBα degradation.

## Materials and Methods

### Cell Culture and Reagents

Human embryonic kidney 293T cells (HEK293T) were grown in Dulbecco’s modified essential medium (DMEM; Invitrogen, USA) containing 10% fetal bovine serum (Gibco), 100 U/mL penicillin (GENVIEW) and 10 μg/mL streptomycin sulfate (GENVIEW) at 37°C in a humidified 5% CO2 incubator. Recombination human TNFα (300-01A) was purchased from PERPROTECH Inc. (Rocky Hill, USA). Dimethyl sulfoxide (DMSO, ST038) was purchased from Beyotime Biotechnology Inc. ANTI-FLAG M2 Affinity Gel (A2220) was obtained from SIGMA. Protein A/G plus-agarose (sc-2003) was obtained from Santa Cruz. 3×FLAG peptide (F7499) was purchased from SIGMA.

### Constructs

All TRIM-expression plasmids pTRIP-TRIMs-3FLAG-RFP used in this study were stored in our laboratory (**Table S1**). Various mutated constructs of TRIM67 (TRIM67SA, ΔR, ΔN, ΔC) were cloned into lentiviral expression vector pTRIP-3FLAG -RFP (33). Full-length TRIM67 were cloned into vector pLVX-EF1α-EGFP. Full-length β-TrCP and IκBα were obtained by polymerase chain reaction (PCR) from 293T cDNA using specific primer (**Table S2**). Wild type and deleted constructs of β-TrCP (β-TrCP.N and β-TrCP.C) were cloned into pcDNA3.1-HA. Wild type construct of IκBα was cloned into vector pCMV-C-Myc. All constructs were confirmed through DNA sequencing.

### Antibodies

Mouse monoclonal antibodies against FLAG-tag (M185-3L) and HA-tag (M180-3) were purchased from MEDICAL & BIOLOGICAL LABORATORIES CO., LTD. (MBL, Japan). Antibody against Myc-tag (16286-1-AP) was purched from Proteintech Group Inc. (China). Rabbit anti-RELA polyclonal antibodies (A2547) and FITC-conjugated goat anti-mouse antibodies were obtained from ABclonal Biotech Co., Ltd (USA). Rabbit anti-IκBα polyclonal antibody (10268-1-AP), rabbit anti-β-TrCP polyclonal antibodies (13149-1-AP), and mouse anti-alpha tubulin monoclonal antibodies (66031-1-Ig) were purchased from Proteintech Group Inc. (China). Horseradish peroxidase-conjugated (HRP) goat anti-mouse and goat anti-rabbit IgG (H+L) secondary antibodies were obtained from Boster Bioengineering Ltd (China).

### Lentivirus Particle Production

All TRIM plasmids were used to generate lentivirus stocks in conjunction with helper plasmids pCMV-gag-pol and pCMV-VSVg (34). Briefly, 293T cells in 6-well plates were co-transfected with pTRIP-TRIMs-3FLAG (1.0 μg) plus pCMV-gag-pol (0.8 μg) and pCMV-VSVg (0.2 μg) using Lipofectamin 2000. Lentivirus particles were collected at 48 h post-transfection.

### Immunofluorescence Assay

293T cells were seeded in 48-well plates and transduced with lentivirus-expressing TRIMs proteins. At 48 h post-transduction, cells were treated with or without TNFα (50 ng/mL) for 30 min. Cells were fixed in 4% paraformaldehyde for 20 min and permeabilized with 0.1% Triton X-100 at room temperature (RT) for 10 min. The cells were then washed three times with phosphate-buffered saline (PBS) and then incubated with rabbit anti-p65 antibodies at RT for 2 h. After three washes with PBS, the cells were incubated with FITC-conjugated goat anti-rabbit antibodies at RT for 1 h. The cells were washed again three times with PBS before incubation with 4′, 6-diamidino-2-phenyl-indole (DAPI, Sigma) for 15 min. Fluorescent images were obtained using microscope.

### Western Blotting

Cells were lysed with NP40 lysis buffer (1.19% HEPES, 0.88% NaCl, 0.04% EDTA, 1% NP40 and a protease inhibitor (Roche, UK)). The cytoplasmic and nuclear proteins were fractioned using NE-PER Nuclear and Cytoplasmic Extraction Kit (Thermo Fisher Scientific, Cat#78833). The protein concentration of whole-cell lysates was determined using bicinchoninic acid protein assay kit (Thermo Scientific) to evaluate protein expression. Equal amounts of proteins were separated using 12% sodium dodecyl sulfate polyacrylamide gels (SDS-PAGE) and transferred onto polyvinylidene fluoride (PVDF) membranes (Roche, UK). The membranes were blocked with 5% non-fat milk in 1×Tris-buffered saline (TBS) with 5% Tween-20 (DGBio, Beijing, China) for 4 h at room temperature (RT). The membranes were subsequently incubated with diluted primary antibodies at RT for 2 or at 4°C overnight. Anti-rabbit or anti-mouse IgG antibodies conjugated to HRP were used as secondary antibodies. An enhanced chemiluminescence substrate was used in detecting by HRP kit (Thermo Scientific, USA). All immunoblot images were performed using Bio-Rad ChemiDoc XRS+ instrument and image software.

### Immunoprecipitation and LC-MS/MS

Cells seeded in 60 mm dish were transected with pTRIP-TRIM67-3FLAG plasmid or empty vector 3.0 μg. At 30 h post-transfection, cells were collected and lysed with NP40 lysis buffer containing protease inhibitor in ice for 30 min. Samples were subjected to centrifugation for 10 min at 4°C to remove cellular debris. Cell lysates were incubated with anti-FLAG M2 affinity gel in rolling incubator at 4°C overnight. Lysates were discarded after a brief centrifugation at 3,000 g for 5 min at 4°C. The beads were washed five times with cold lysis buffer prior to elution using 3X FLAG peptide. The solution was performed LC-MS/MS analysis to identify TRIM67 interaction proteins by Shanghai Applied Protein Technology. When protein A/G plus-agarose (Santa Cruz) was used for Co-immunoprecipitation (Co-IP), the cells lysates were incubated with indicated antibodies at 4°C for 5 h firstly. At 5 h later, samples were subjected to centrifugation at 3,000 g for 1 min at 4°C. Then carefully transfer the supernatant into Protein-A/G plus-agarose that has been washed with lysis buffer. Samples were incubated in rolling incubator at 4°C for 4 h. Supernatants were discarded after a brief centrifugation at 3,000 g for 5 min at 4°C. The beads were washed five times with cold lysis buffer prior to elution by incubation at 95°C in 1× sample buffer.

### NF-κB Luciferase Activity Assay

293T cells were seeded in 24-well plates 24 h prior to transfection. Cells were co-transfected with a reporter plasmid encoding NF-κB -Luc plus pTK-*Renilla* and wild type and mutated TRIM67 expression plasmid. The empty vector was used as negative control to adjust for the total amount of transfected DNA. At 24 h post-transfection, cells were treated with or without TNFα (10 ng/mL) for 10 h. The firefly and *Renilla* luciferase activities were determined using Dual-Luciferase Reporter Assay System (E1910, Promega, Madison, USA), in accordance with to the manufacturer’s instructions. Firefly luciferase was normalized to *Renilla* luciferase readings in each well, and data were plotted as fold change relative to that of the empty vector for at least three triplicate experiments carried out on separate days. All reporter assays were repeated three times. Data are presented as mean ± standard deviations (SD).

### Real-Time PCR Analysis

293T cells were transfected with indicated plasmid. At 24 h post-transfection, cells were treated with or without TNFα (10 ng/mL) for 6 h. Cellular total RNA was extracted with the TRIzol reagent (Invitrogen, Grand Island, NY, USA) in accordance with the manufacturer’s instructions. Total RNA (1.0 μg) was reverse transcribed using First-Strand cDNA Synthesis Kit (TOYOBO) according to the manufacturer’s instructions. The mRNA levels of TNFα and IL-6 genes were determined through relative quantitative real-time PCR using a SYBR Green Real-time PCR Master Mix (TOYOBO) with specific primer pairs: TNFα (5′-CCGAGTGACAAGCCTGTAG-3′ and 5′-GGTCTGGTAGGAGACGGCG-3′), IL-6 (5′-CCAGGAGCCCAGCTATGAAC-3′ and 5′-CTGAGATGCCGTCGAGGATG-3′). The cycling conditions were 95 °C for 10 min, followed by 40 cycles at 95 °C for 30 s, at 56 °C for 30 s, at 72 °C for 30 s. All reactions were performed in triplicate and the mRNA level of the housekeeping gene GAPDH was used as endogenous reference control.

### Statistical Analysis

All experiments were performed at least three different biological replicates, all replicates show similar results. One of three replicates was presented in the manuscript. GraphPad Prism software Version 5 (GraphPad Prism Version 5, GraphPad Software, La Jolla, CA, USA, 2012) was used in this study. The various treatments were compared using an unpaired, two-tailed Student’s t-test with an assumption of unequal variance. P < 0.05 was considered statistically significant. In addition, P < 0.01 and P < 0.001 were marked with two (**) and three (***) asterisks, respectively.

## Results

### TRIM67 Suppresses TNFα-Triggered NF-κB Activation

**Figure 1:**
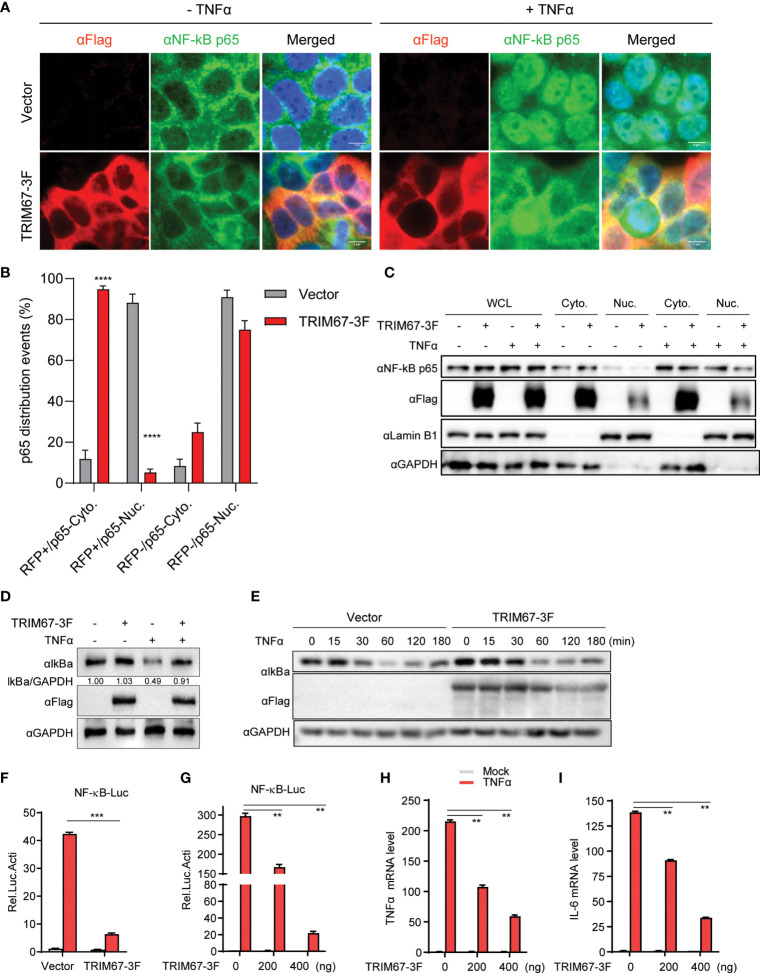
TRIM67 negatively regulates TNFα-triggered NF-κB activation. **(A)** 293T cells were transduced with lentivirus expressing TRIM67 or blank control lentivirus. At 48 h post-transduction, the cells were treated with or without TNFα (50 ng/mL) for 20 min. Cells were fixed and stained using anti-p65 antibodies and DAPI. Images were obtained using confocal microscopy. Red, anti-FLAG -stained cells; green, anti-p65-stained cells; blue, DAPI-stained cells. Scale bar: 5 µm. **(B)** The events of p65 nuclear translocation in TRIM67-expressing cells or empty vector-expressing cells that treated with TNFα for 30 min. **(C)** HeLa cells were transfected with TRIM67-3F-expressing plasmids or empty vector. At 48 h post-transduction, cells were treated with or without TNFα (50 ng/mL) for 30 min, then cells were collected for cytoplasmic and nuclear extraction in accordance with the manufacturer’s instruction. Western blot was performed to detect indicated proteins using specific antibodies. **(D)** 293T cells were transduced with lentivirus expressing TRIM67 or blank control lentivirus. At 48 h post-transduction, cells were treated with or without TNFα (50 ng/mL) for 30 min. The protein levels of IκBα were measured with Western blot using anti-IκBα antibodies. **(E)** Western blot analysis of the protein levels of endogenous IκBα from 293T cells transfected plasmids expressing TRIM67-3F and treated with TNFα (10 ng/mL) for the indicated time points. **(F, G)** 293T cells were transfected with *Renilla* luciferase and NF-κB luciferase reporter constructs plus 400 ng TRIM67-expressing plasmid (**G**: increasing amount of TRIM67-expressing plasmids) an empty vector. At 24 h post-transfection, cells were treated with or without TNFα (10 ng/mL). Luciferase activities were measured at 10 h post-treatment. **(H, I)** 293T cells were transfected with plasmids expressing TRIM67 or empty vector, followed by TNFα treatment for 6 hours. The mRNA expression levels of TNFα **(H)** and IL-6 **(I)** were analyzed by Real-time PCR assay using specific primer pairs. Endogenous GAPDH mRNA served as control. P < 0.05 was considered statistically significant. In addition, P < 0.01, P < 0.001, and P < 0.0001 were marked with two (**), three (***), and four (****) asterisks, respectively.

To investigate the potential capability of TRIM proteins to regulate NF-κB signaling pathways, we established a microscopy-based assay and subsequently identified the effects of TRIM proteins on TNFα-triggered NF-κB activation. The screening was based on the microscopy observation of the nuclear translocation of p65 that was induced by TNFα. We examined the effects of 22 human TRIM proteins on the TNFα-triggered nuclear translocation of p65. We found that the TNFα-triggered nuclear translocation of p65 was significantly restricted by TRIM67 expression (**Table S1**). As shown in **Figure 1A**, TRIM67 expression exerted no effects on the cellular localization of p65 in the absence of TNFα. By the analysis of p65 nuclear translocation events, we found that 95% of TRIM67-expressing cells showed p65 cytoplasmic retention with the treatment of TNFα (**Figure 1B**). However, the TNF-triggered nuclear translocation of p65 was significantly inhibited in the presence of TRIM67. Furthermore, this effect of TRIM67 was confirmed by cellular fractionation assay of p65 (**Figure 1C**).

The release of the NF-κB subunit p65 from IκBα/p65:p50 complex and its translocation from the cytoplasm to the nucleus are critical to activate NF-κB-mediated gene expression (2). To confirm the inhibition ability of TRIM67, a NF-κB promoter-mediated luciferase activity was conducted by co-transfection of NF-κB promoter reporter with plasmids-expressing TRIM67 or an empty vector, then, the cells were treated with or without TNFα. We found that the overexpression of TRIM67 exerts no effect on IκBα protein level change in the absence of TNFα, but it significantly blocked the TNFα-induced degradation of IκBα (**Figure 1D**). As further confirmation, TNFα-triggered IκBα degradation was delayed in the presence of TRIM67 expression (**Figure 1E**). Next, NF-κB promoter reporter assay was performed to evaluate TRIM67’s inhibition. As shown in **Figure 1F**, TNFα-triggered NF-κB activity was significantly repressed in the expression of TRIM67. Moreover, the inhibitory ability of TRIM67 was in an amount-dependent manner (**Figure 1G**). Finally, we also found that the expression of TRIM67 significantly inhibited the NF-κB-dependent expression of the pro-inflammatory cytokines TNFα and IL-6 (**Figures 1H, I**).

### Knockout Trim67 Elevates TNFα-Triggered Inflammatory Response in Mouse Primary Cells

**Figure 2:**
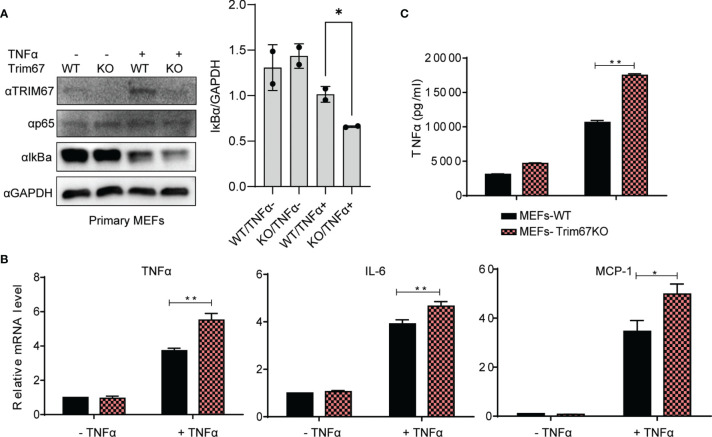
Trim67-defficiency promoted inflammatory cytokine production in MEF cells derived from Trim67 knockout mice. **(A)** Left: Embryonic fibroblasts from WT and Trim67-knockout mice were treated with or without TNFα, then the protein levels of IκBα, p-p65, Trim67, and GAPDH were analyzed by Western blot using the indicated antibodies. Right: the ratio of IκBα to GAPDH in the individual conditions. **(B)** WT or Trim67-knockout MEFs were stimulated with or without TNFα (50 ng/mL). The mRNA expression levels of TNFα, IL-6, and MCP-1 were analyzed by Real-time PCR. Endogenous GAPDH mRNA served as control. **(C)** WT or Trim67-knockout MEFs were stimulated with or without TNFα (50 ng/mL). The protein levels of TNFα were detected by using MSD U-PLEX Biomarker Group 1 (mouse) Multiplex Assay. P < 0.05 was considered statistically significant. In addition, P < 0.05 and P < 0.01 were marked with one (*) and two (**) asterisks, respectively.

To evaluate the physiological function of TRIM67 in regulating NF-κB signaling pathway, we investigated its effects by applying mouse embryonic fibroblasts (MEFs) from wild-type mice and Trim67 knockout mice. The wild-type and Trim67 knockout MEF cells were treated with or without TNFα, followed Western blot analysis of IκBα protein degradation. As shown in **Figure 2A**, Trim67 deficiency promotes TNFα-triggered IκBα degradation. We also found that few NF-κB signaling pathway dependent inflammatory cytokines, such as TNFα, IL-6, and Mcp-1 were upregulated in Trim67 knockout MEFs with TNFα treatment (**Figure 2B**). Furthermore, we also observed that the increased protein expression of TNFα along with TNFα-treatment in Trim67 deficient MEFs (**Figure 2C**). Together, our findings demonstrate that TRIM67 is novel negative regulator of TNFα-dependent NF-κB activation.

### TRIM67 Inhibits NF-κB Activation by Acting in the Level Between IκBα and p65

**Figure 3:**
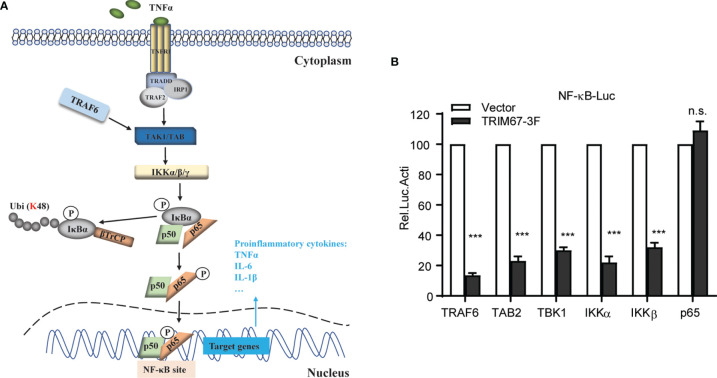
TRIM67 inhibits TNFa-mediated NF-kB activation at the level between IKKs and p65. Identification of the action stage of TRIM67. **(A)** Schematic of TNFα-triggered NF-κB signaling pathway. After binding the extracellular TNFα with the TNF receptors (TNFR), TRADD, RIP, and TRAFs were recruited to the oligomeric TNFR complexes to induce the activation of the TAK1, TAB, and IKKs complexes. Subsequently, the activated IKKs phosphorylated IκBα, thus leading to its ubiquitination and degradation and resulting in the activation of NF-κB. **(B)** 293T cells were co-transfected with Renilla luciferase and NF-κB firefly luciferase reporter constructs plus 400 ng of TRIM67 expression plasmid (400 ng) or NF-κB activators TRAF6, TAB2, TBK1, IKKα, IKKβ, and p65 (400 ng). At 24 h post-transfection, cells were treated with or without TNFα (10 ng/mL). Luciferase activities were measured at 10 h post-treatment. P < 0.001 was marked with three (***) asterisks. ns, not significant.

Upon TNFα stimulation, the activated IKK kinase complex mediated the phosphorylation of IκBα. Subsequently, the phosphorylated IκBα proteins were degraded through the 26S-proteasome pathway. Finally, the freed NF-κB was transported into the nucleus to activate the transcription of numerous genes (**Figure 3A**). To determine the stage at which the action of TRIM67 occurred, we co-transfected the NF-κB reporter with several known NF-κB mediators, including TRAF6, TAB2, TBK1, IKKα, IKKβ, and p65 with TRIM67, or an empty vector into 293T cells. The results showed that the TRIM67 expression significantly repressed the NF-κB activation induced by RAF6, TAB2, TBK1, IKKα, and IKKβ. However, TRIM67 expression exerts no effect on the p65-mediated NF-κB activation (**Figure 3B**). Hence, TRIM67 acted at the upstream of p65 and at the downstream of the IKK complex.

### The Interaction Between TRIM67 and β-TrCP Is Required for TRIM67-Mediated Inhibition of NF-κB

**Figure 4:**
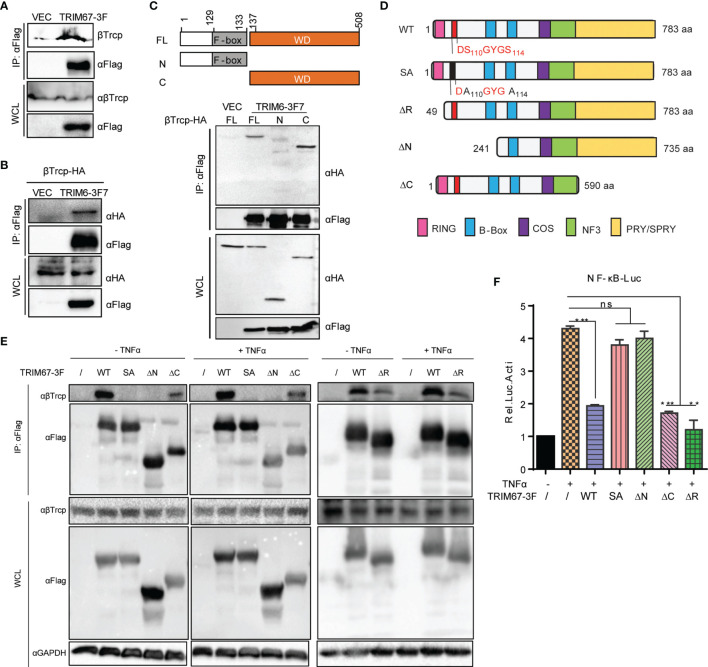
The amino acid residues S110 and S114 of TRIM67 are critical for its interaction with β-TrCP and subsequential NF-κB inhibition. **(A)** 293T cells were transfected with TRIM67-FLAG expressing plasmid or an empty vector. At 30 h post-transfection, the cell lysates were immunoprecipitated using anti-FLAG affinity gel then detected with antibodies against FLAG -tag and β-TrCP. **(B)** 293T cells were co-transfected with HA-tagged β-TrCP and TRIM67-FLAG expressing plasmid or an empty vector. At 30 h post-transfection, the cell lysates were immunoprecipitated using anti-FLAG affinity gel and then detected with antibodies against FLAG-tag and HA-tag. **(C)** Top panel: Schematic representation of β-TrCP domains and the individual β-TrCP mutants used in this study. Bottom panel: Plasmids expressing full length FLAG-tagged TRIM67 or an empty vector were co-transfected with wild type or truncated HA-tagged β-TrCP constructs (top panel) into 293T cells. At 30 h post-transfection, the cell lysates were immunoprecipitated using anti-HA antibodies and then enriched with protein A/G plus-gel. After washing five times, the samples were analyzed with Western blot analysis using antibodies against FLAG-tag and HA-tag. **(D)** Schematic representation of TRIM67 domains and the individual TRIM67 mutants used in this study. **(E)** 293T cells were transfected with wild type and individual mutated TRIM67. At 48 h post-transfection, the cells were treated with or without TNFα for 30 min. The interaction between TRIM67 and endogenous β-TrCP was determined by Co-IP assay using antibody anti-FLAG tag. **(F)** 293T cells were transfected with *Renilla* luciferase and NF-κB luciferase reporter constructs plus wild type and mutated TRIM67-expressing plasmid (400 ng) and an empty vector. At 24 h post-transfection, the cells were treated with or without TNFα (10 ng/mL). Luciferase activities were measured at 10 h post-treatment. In (F), P < 0.01 and P < 0.001 were marked with two (**) and three (***) asterisks, respectively. P > 0.05 was marked ns indicates there is not statistically significant.

To investigate whether TRIM67 interacts with certain regulatory molecules in NF-κB pathways, we tested the interaction between TRIM67 and TRAF6, TAB2, TBK1, IKKα, IKKβ, IκBα, and p65 by using co-immunoprecipitation. However, we failed to observe TRIM67 interacting with any molecules (data not shown). To discover the potential mechanisms of the TRIM67-mediated suppression of NF-κB activation, we performed an immunoprecipitation combined with mass spectrometry analysis and later identified a TRIM67 interaction protein in the 293T cells. β-transducin repeat-containing protein (β-TrCP, also as known as FBXW11) was identified as a TRIM67 interaction protein. β-TrCP plays a central role in the regulation of NF-κB activation by targeting IκBα for proteasomal degradation (9, 35). Furthermore, we confirmed the interaction between TRIM67 and β-TrCP through immunoprecipitation. The results showed that FLAG-tagged TRIM67 can precipitate HA-tagged β-TrCP (**Figure 4A**), as well as endogenous β-TrCP (**Figure 4B**). To determine the interaction motif between TRIM67 and β-TrCP, we established two truncated constructs of β-TrCP with the deletion of C-terminal WD40 repeat region of N-terminus (**Figure 4C**, topper). We found that β-TrCP interacted with TRIM67 through its WD40 repeat region (**Figure 4C**, bottom). On the other hand, the interaction motif of TRIM67 was determined by using different TRIM67 mutants (**Figure 4D**). As shown in **Figure 4E**, the N-domain-deleted TRIM67 negated the interaction between TRIM67 and β-TrCP. Notably, two amino acid residues S110 and S114 contribute to TRIM67 interacting with β-TrCP.

Next, we investigated the connection between TRIM67 and β-TrCP to TRIM67-mediaed inhibition of TNFα-triggered NF-κB activation. To do so, we evaluated the inhibitory ability of the different mutants of TRIM67 to suppress TNFα-triggered NF-κB activity by using NF-κB promoter activity assay. As shown in **Figure 4F**, corresponding to TRIM67-β-TrCP interaction (**Figure 4E**), the mutant of TRIM67, such as S110/114A and N-terminus deleted truncation (ΔN) that failed to interact with β-TrCP failed to inhibit TNFα-triggered NF-κB activation. We found that the interaction between TRIM67 mutant that with RING domain deletion (TRIM67ΔR) and β-TrCP was attenuated. Whereas TRIM67ΔR still significantly suppresses TNFα-triggered NF-κB activation. Overall, these results suggested that the interaction between TRIM67 and β-TrCP is important for TRIM67-mediated suppression of NF-κB.

### TRIM67 Competitively Binds β-TrCP to IκBα

**Figure 5:**
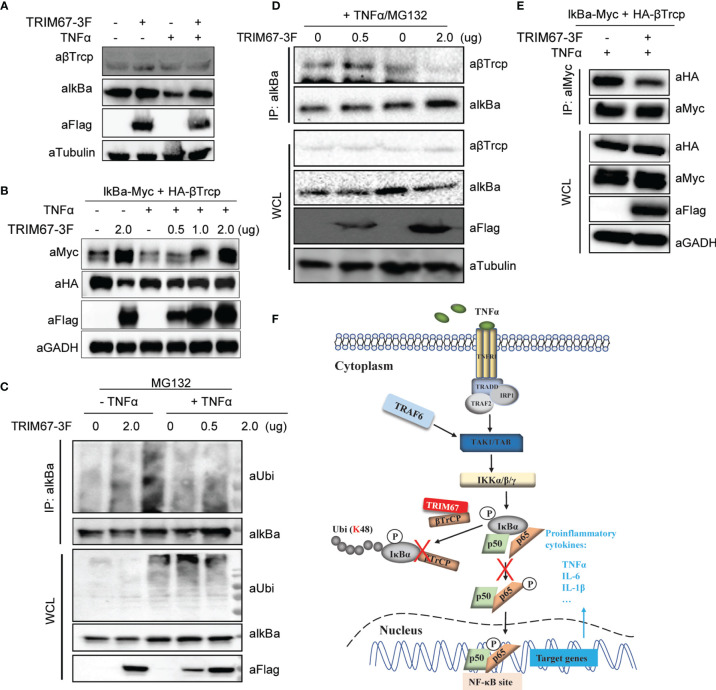
TRIM67 attenuates the interaction between β-TrCP and IκBα. **(A)** 293T cells were transfected with TRIM67-expressing plasmids or an empty vector. At 24 h post-transfection, cells were treated with or without TNFα (50 ng/mL) for 30 min. Cells were harvested, and the equal amounts of cell lysates were analyzed with Western blot using antibodies against IκBα, β-TrCP, and FLAG-tag. **(B)** Plasmids expressing Myc-tagged IkBa and HA-Tagged β-TrCP were co-transfected with TRIM67-3F expressing plasmids into 293T cells. At 24 h post-transfection, cells were treated with or without TNFα for 30 min, then followed Western blotting assay using the indicated antibodies. **(C)** 293T cells were transfected with plasmids expressing TRIM67-FLAG and an empty vector. At 24 h post-transfection, cells were treated with TNFα (50 ng/mL) in the presence of MG132 (20 μM) for 30 min. Cells were harvested, and equal amounts of cell lysates were precipitated using antibodies against IκBα. The results were analyzed with Western blot using antibodies against IκBα, β-TrCP, FLAG-tag, and tubulin. **(D)** 293T cells were transfected with TRIM67-expressing plasmids or an empty vector. At 24 h post-transfection, cells were treated with or without TNFα (50 ng/mL) in the presence of MG132 (20 μM) for 30 min. Cells were harvested, and equal amounts of cell lysates were precipitated using antibodies against IκBα. Subsequently, the results were analyzed with Western blot using antibodies against IκBα, ubiquitin, FLAG-tag. **(E)** Plasmids expressing Myc-tagged IkBa and HA-Tagged β-TrCP were co-transfected with TRIM67-3F expressing plasmids into 293T cells. At 24 h post-transfection, cells were treated with or without TNFα for 30 min, then followed Co-IP assay using antibody against Myc-tag. **(F)** A proposed model describing the role of TRIM67 in the regulation of TNFα-mediated NF-κB activation. Upon the extracellular TNFα bond TNF receptors (TNFR), TRADD, RIP, and TRAFs were recruited to the oligomeric TNFR complexes to induce the activation of the TAK1, TAB, and IKKs complexes. Subsequently, the activated IKKs phosphorylated IκBα. Next, the phosphorylated IκBα was ubiquitinated by E3 ubiquitin ligase β-TrCP for proteasomal degradation, which resulted the phosphorylation and translocation of p50/p65 to activate NF-κB signaling pathway. However, in the presence of TRIM67, TRIM67 competitively binds to β-TrCP to limit it-mediated proteasome degradation of IκBα. As a result, the TNFα-triggered activation of NF-κB signaling pathway was inhibited by TRIM67.

The β-TrCP-mediated degradation of IκBα is a critical action in NF-κB signaling pathways (5). To demonstrate how TRIM67 regulates NF-κB activation through its interaction with β-TrCP, we first investigated the effect of TRIM67 on the stabilization of the endogenous β-TrCP protein. 293T cells were transfected with expression plasmids of 3×FLAG -tagged TRIM67 or an empty vector. At 24 h post-transfection, the cells were treated with or without TNFα (50 ng/mL) for 30 min. The results showed that the TRIM67 has no effects on the stabilization of β-TrCP in the presence or absence of TNFα (**Figure 5A**). Next, we co-overexpressed Myc-tagged IκBα and HA-tagged β-TrCP with 3FLAG-tagged TRIM67, the cells were treated with or without TNFα. We found that TRIM67 has no effects on the protein levels of exogenous β-TrCP. Whereas TNFα-induced degradation of IκBα-Myc was suppressed by TRIM67 (**Figure 5B**). TRIM9 is known to compete with IκBα for β-TrCP binding to inhibit β-TrCP-mediated IκBα degradation (21). To address whether TRIM67 functions similarly to TRIM9, we firstly examined the effects of TRIM67 on TNFα-induced ubiquitination of IκBα. We found that IκBα proteins were significantly ubiquitylated upon TNFα treatment in the absence of TRIM67. In contrast, its ubiquitylation was markedly suppressed in the presence of TRIM67 expression (**Figure 5C**). Next, the effects of TRIM67 on the interaction between IκBα and β-TrCP was investigated. 293T cells were transfected with 3×FLAG-tagged TRIM67-IκBα expressing plasmids or an empty vector. At 24 h post-transfection, the cells were treated with TNFα (50 ng/mL) in combination with MG132 for 30 min. Thereafter, the interaction between the endogenous IκBα and β-TrCP was detected. The results showed that the interaction of IκBα with β-TrCP was attenuated in the presence of TRIM67 expression (**Figure 5D**). Again, consistent results were observed in ectopic expression of IκBα and β-TrCP (**Figure 5E**). Collectively, our findings suggest that TRIM67 is novel negative regulator of NFκB signaling pathway, which competitively binding β-TrCP to ubiquitinated IκBα. As a consequential results, the stabilized IκBα keeps inhibiting NFκB activation (**Figure 5F**).

## Discussion

NF-κB is an important transcription regulator that plays a critical role in the regulation of the expression of numerous genes, including pro-inflammatory cytokines, chemotactic factors, growth factors, and effector enzymes (1, 4). NF-κB is associated with many cellular events such as immune responses (36), cancer (37–40), and inflammatory diseases (41–43). Upon viral infection, NF-κB is manipulated by viral proteins through targeting host pattern recognition receptors (PRRs) and certain molecules (44, 45). Virus-modulated NF-κB activation is associated with both infectious diseases and innate immune response. Owing to its constitutive activation, NF-κB plays a critical role in the maintenance and expansion of cancer stem cells in most tumors. The inhibitor of NF-κB could potentially facilitate tumor therapy (40, 46). In addition, NF-κB activation is involved in many inflammatory diseases. Evidence suggests that the careful use of the inhibitors of NF-κB activation could result in the control of some chronic inflammatory diseases (42). And some studies reported that medicines were used to block NF-κB subunits gene expression and then inhibit neuroinflammation mediator production or IL-1β-Induced inflammatory responses (47, 48). In summary, the careful regulation of NF-κB activation is crucial for host health and disease control.

Previous studies revealed that β-TrCP plays a critical role in the recognition and degradation of phosphorylated IκBs in the regulation of the activation of NF-κB pathways (9, 35). In addition, β-TrCP positively regulates TAK1-dependent NF-κB activation by targeting interleukin-1 (IL-1) receptor-associated kinase (IRAK1) for degradation to release the TAK1-TRAF6 complex from the membrane to the cytosol (49). Owing to the central role of β-TrCP in NF-κB pathways, β-TrCP can be targeted for modulating the activation of NF-κB. Vaccinia virus inhibits NF-κB activation through the viral protein A49 that binds with β-TrCP and thereby suppresses IκBα degradation (50). Human rotaviruses target β-TrCP for degradation *via* the viral nonstructural protein 1, resulting in the inhibition of NF-κB activation (51–53). Rotaviruses can also target β-TrCP to evade host innate immune response and suppress other cellular activities. In addition, histidine triad nucleotide-binding protein 1 (HINT1), a novel tumor suppressor stabilizes IκBα protein levels by targeting β-TrCP (54).

Emerging evidence suggests that TRIM proteins play an important role in the regulation of NF-κB-dependent inflammation (17). Most studies have revealed that TRIM proteins mediate NF-κB pathways by ubiquitinating NF-κB-related adaptor proteins, kinase proteins, and transcriptional factors (17). Some TRIM proteins, such as TRIM4 and TRIM25, serve as positive regulators of NF-κB pathways. Specifically, they target RIG-I for K-63-linked poly-ubiquitination, thereby activating RIG-I-mediated NF-κB activation (55, 56).. By contrast, some TRIM proteins target the kinase protein TAB2/3 and IKK complex to display the potential inhibitory function of NF-κB signaling (18–20, 57–59). In addition, other TRIM proteins, including TRIM9 and TRIM39, can target another NF-κB-associated regulator, such as β-TrCP and cactin, to modulate NF-κB signaling (21, 60). In the present study, TRIM67 was found to be a suppressor of NF-κB signaling pathways. TRIM67, also known as TRIM9-like, is selectively expressed in the cerebellum (26). In a previous study, Shi reported that TRIM9 hijacks β-TrCP to prevent the β-TrCP-mediated degradation of IκBα and p100, thus blocking NF-κB activation (21). They demonstrated that degron motif phosphorylation is required for TRIM9 function; the substitution of serine residues in the degron motif for alanine negates the suppression of TRIM9. Similarly, in TRIM67, its degron motif S110 and S114 is required for interacting b-TrCP (**Figure 4E**). Furthermore, we also demonstrated that TRIM67-meidated inhibition of NF-κB activation is dependent on TRIM67-β-TrCP interaction (**Figure 4F**).

In summary, we identified that TRIM67 as a negative regulator of NF-κB activation by preventing β-TrCP-mediated IκBα degradation. Further studies are needed to examine the effects of TRIM67 on NF-κB signaling regulation by *in vivo* studies in a model lacking TRIM67. Altogether, our findings provide a new insight into the emerging role of TRIM family proteins in regulation of inflammatory response.

## Data Availability Statement

The original contributions presented in the study are included in the article/**Supplementary Material**. Further inquiries can be directed to the corresponding author.

## Author Contributions

WF conceptualized, performed the experiments, analyzed the data, and wrote the manuscript. XLiu, JZ, LQ, and JD performed the experiments and analyzed the data. SQ provided resources. XLi and HC reviewed and edited manuscript. PQ acquired the finding and supervised the study. All authors contributed to the article and approved the submitted version.

## Funding

This work was supported by the National Natural Science Foundation of China (NSFC) (31772713 and 31572495).

## Conflict of Interest

The authors declare that the research was conducted in the absence of any commercial or financial relationships that could be construed as a potential conflict of interest.

## Publisher’s Note

All claims expressed in this article are solely those of the authors and do not necessarily represent those of their affiliated organizations, or those of the publisher, the editors and the reviewers. Any product that may be evaluated in this article, or claim that may be made by its manufacturer, is not guaranteed or endorsed by the publisher.
