# Supplementary material for: TRIM67 Suppresses TNFalpha-Triggered NF-kB Activation by Competitively Binding Beta-TrCP to IkBa
Source: Front Immunol. 2022 Feb 22;13:793147. doi: 10.3389/fimmu.2022.793147 (PMC8901487; doi:10.3389/fimmu.2022.793147)
Supplement: Supplementary file 1 [file Table_1.docx]

**Table 1. TRIM proteins used in the study and their effects on TNFα-triggered nuclear translocation of p65**

| **TRIM** | **GenBank NO.** | **Inhibitory** | **TRIM** | **GenBank NO.** | **Inhibitory** |
| --- | --- | --- | --- | --- | --- |
| TRIM7 | NM_203293 | - | TRIM43 | NM_138800 | - |
| TRIM11 | NM_145214 | - | TRIM44 | NM_017583 | - |
| TRIM14 | NM_014788 | - | TRIM45 | NM_025188 | - |
| TRIM15 | NM_033229 | - | TRIM49 | NM_020358 | - |
| TRIM17 | NM_001024940 | - | TRIM50 | NM_178125 | - |
| TRIM25 | NM_005082 | - | TRIM60 | NM_001258025 | - |
| TRIM26 | NM_003449 | - | TRIM61 | NM_001012414 | - |
| TRIM35 | NM_015066 | - | TRIM62 | NM_018207 | - |
| TRIM37 | NM_001005207 | - | TRIM67 | NM_001004342 | + |
| TRIM39 | NM_021253 | - | TRIM68 | NM_018073 | - |
| TRIM40 | NM_138700 | - | TRIM74 | NM_198853 | - |
