# Supplementary material for: TRIM67 Suppresses TNFalpha-Triggered NF-kB Activation by Competitively Binding Beta-TrCP to IkBa
Source: Front Immunol. 2022 Feb 22;13:793147. doi: 10.3389/fimmu.2022.793147 (PMC8901487; doi:10.3389/fimmu.2022.793147)
Supplement: Supplementary file 2 [file Table_2.docx]

**Table 2. Primers used in the study**

| **Gene** | **GenBank**  **NO.** | **Primer sequence（5’ to 3’）** | **product（bp）** |
| --- | --- | --- | --- |
| GAPDH  TNFα  IL-6  IL-1β  IP-10  IFN-γ  MCP-1  cTRIM67  qmTRIM67  TRIM67  TRIM67ΔR  TRIM67SA  TRIM67ΔN  TRIM67ΔC  βTrCP  βTrCP.N | NM_002046  NM_000594  NM_000600  NM_008361  NM_021274  NM_008337  NM_011333  NM_198632  NM_198632  NM_001004342  NM_001004342  NM_001004342  NM_001004342  NM_001004342  NM_033645  NM_033645 | F: GAGTCAACGGATTTGGTCGT | 239  246  210  116  131  163  125  780  391  205  2352  2211  2352  1635  1770  1539  423 |
|  |  | R: TTGATTTTGGAGGGATCTCG  F: CCGAGTGACAAGCCTGTAG  R: GGTCTGGTAGGAGACGGCG  F: CCAGGAGCCCAGCTATGAAC  R: CTGAGATGCCGTCGAGGATG  F: GAAATGCCACCTTTTGACAGTG  R: TGGATGCTCTCATCAGGACAG  F: CCTGCTGGGTCTGAGTGGGA  R: GATAGGCTCGCAGGGATGAT  F: GACTGTGATTGCGGGGTTGT  R: GGCCCGGAGTGTAGACATCT  F: CGGAACCAAATGAGATCAGAA  R: TGTGGAAAAGGTAGTGGATGC  F1: GATGATAGCCATGTAATGCCCACC  F2: TGCCGTTTTCCCCTTCTAAATCAG  R: CCGTGATATGCTTGCCACAGGTTC  F: GCTCACCAAGGTGACCAAAG  R: CTTTGACCCACTGCTCTTGC  F:ACTAGTATGGAGGAAGAGCTGAAGTGTCC  R:GTCGACCTGATTATAGGCCTTGCTG  F:ACTAGTATGCTGCCTTGCGCTCGCACCATC  F:GACAGACGCTGGCTACGGGGCTTACACCCCGAGCCTCAAGTCCC  F: ACTAGTATGTCCCGGGGACCCTTCGCCAAG  R:GTCGACGAAAGCTTTGACTCGGGCGTTG  F: GAATTCATGGAGCCCGACTCGGTGATTGAG  R:GTCGACCTATCTAGAGATGTAAGTGTATGTTCTG  R: GTCGACCTATCGTTCAATCAGCTTCTTCCAAAGC |  |
| βTrCP.C | NM_033645 | F: GAATTCATGGTACGCACTGATCCCCTATGGAAAGG | 1131 |

F: Forward, R: Reverse. Bold, the substitution of Serine with Alanine. Underline, restriction endonucleases recognition sequences.
